# DIETARY RESTRICTION AND THE TRANSCRIPTION FACTOR CLOCK DELAY EYE AGING TO EXTEND LIFESPAN IN DROSOPHILA MELANOGASTER

**DOI:** 10.1093/geroni/igad104.1680

**Published:** 2023-12-21

**Authors:** Pankaj Kapahi

**Affiliations:** Buck Institute for Research on Aging, Novato, California, United States

## Abstract

Many vital processes in the eye are under circadian regulation, and circadian dysfunction has emerged as a potential driver of eye aging. Dietary restriction (DR) is one of the most robust lifespan-extending therapies and amplifies circadian rhythms with age. We have previously shown that lifespan extension upon DR requires the presence of circadian clocks for maximal benefits. Herein, we demonstrate that dietary restriction extends lifespan in Drosophila melanogaster by promoting circadian homeostatic processes that protect the visual system from age- and light-associated damage. Altering the positive limb core molecular clock transcription factor, CLOCK, or CLOCK-output genes, accelerates visual senescence, induces a systemic immune response, and shortens lifespan. Flies subjected to dietary restriction are protected from the lifespan-shortening effects of photoreceptor activation. Inversely, photoreceptor inactivation, achieved via mutating rhodopsin or housing flies in constant darkness, primarily extends the lifespan of flies reared on a high-nutrient diet. Our findings establish the eye as a diet-sensitive modulator of lifespan and indicates that vision is an antagonistically pleiotropic process that contributes to organismal aging.

